# Interleukin 17A as a good predictor of the severity of *Mycoplasma pneumoniae* pneumonia in children

**DOI:** 10.1038/s41598-017-13292-5

**Published:** 2017-10-11

**Authors:** Mingyue Yang, Fanzheng Meng, Kuo Wang, Man Gao, Ruihua Lu, Mengyao Li, Fangxing Zhao, Lijuan Huang, Yining Zhang, Genhong Cheng, Xiaosong Wang

**Affiliations:** 1 Department of Translational Medicine, the First Hospital of Jilin University, Changchun, 130021 China; 2 Department of Pediatrics, the First Hospital of Jilin University, Changchun, 130021 China; 3 The Bethune Medical School of Jilin University, Changchun, 130021 China; 4 Department of Respiratory, Changchun Children’s Hospital, Changchun, 130051 China; 5 Department of Microbiology, Immunology and Molecular Genetics, University of California Los Angeles, Los Angeles, CA 90095 USA

## Abstract

Early distinction between severe *Mycoplasma pneumoniae* pneumonia (MPP) and mild MPP is still difficult. The aim of this study was to analyze cytokines in bronchoalveolar lavage fluid (BALF) and explore predicting factors of severe MPP in children. Retrospective analysis was performed on 150 children with MPP or bronchial foreign body (FB) admitted in our hospital. The mRNA levels of IL17A were found significantly lower in severe MPP group comparing with mild MPP group or FB group. However, no significant difference was found in the levels of IL4, IL10 or interferon beta1 (IFNβ1) between the two groups. Receiver operator characteristic (ROC) curve analysis showed that IL17A can be used to distinguish severe MPP from mild MPP. These results were confirmed in a validation cohort including 40 MPP children from another hospital. IL17A levels were correlated with some clinical characters, such as refractoriness and pleural effusion. Lower IL17A levels were more likely to be found in refractory MPP children or in MPP children with pleural effusion. Moreover, the protein levels of IL17A in BALF were also found greatly decreased in children with severe MPP. Thus, decreased IL17A levels in BALF may be a valuable biomarker to identify severe MPP in children.

## Introduction

*Mycoplasma pneumoniae* pneumonia (MPP) counts for 20 to 40% of community-acquired pneumonia in children and shows an even higher incidence during epidemics^1–3^. Severe cases of MPP often have complications including pulmonary necrosis, pulmonary atelectasis, pulmonary consolidation and respiratory failure, increasing the rate of morbidity, mortality as well as the cost of health care in our society. Mild MPP is usually sensitive to macrolides. However, severe MPP often show clinical and radiological deterioration despite of macrolides therapy for 7 days or longer, glucocorticoid has to be added to treat severe MPP and usually has dramatic beneficial effects^4^. Therefore, in order to prevent the progression of the disease effectively, early diagnosis and screening children of severe MPP is very important for pediatrician to treat it promptly including selection of antibiotics and usage of glucocorticoid.

Some clinical indicators are helpful to diagnose severe MPP. Severe MPP may have pleural effusion, longer fever duration and refractoriness. It has been reported that MP patients with pleural effusion may have a more severe form MPP compared to those without pleural effusion^5^. The mechanism of refractory MPP is not clear, one possible cause is the inflammatory response caused by immune abnormalities. Therefore, glucocorticoid has been used clinically to treat refractory MPP^6–8^. Laboratory findings indicated elevated levels of C-reactive protein (CRP) and lactate dehydrogenase (LDH) in severe MPP patients^9–11^. However, these indicators are nonspecific for severe MPP. Although early diagnosis is very important as to avoid delay of effective treatment, it is still difficult to distinguish severe MPP from mild MPP at the early stage of the disease in children based on all of these indicators.

Considering that biomarkers of severe MPP are produced directly by the immune or non-immune cells in airways as a response to the presence of *Mycoplasma* *pneumoniae* (MP), investigating specific molecular markers in airways might serve as an important adjunct to routine examination for severe MPP diagnosis. The utility of cytokines in bronchoalveolar lavage fluid (BALF) for differential diagnosis of lung cancer has been described in several studies^12,13^. However, up to data, little study has been performed to identify cytokines in BALF as biomarkers of severe MPP. IL17 is a pro-inflammatory cytokine which induces differentiation and migration of neutrophils. Retinoid-related orphan receptor gamma t (RORγt) has been found to induce the transcription of the genes encoding IL17^14^. IL17 is primarily produced by Th17 cells^15^. However, it is increasingly accepted that diverse innate myeloid immune cells are able to produce IL17. These cells are strategically positioned in the barrier tissues, such as lungs, intestines, skin and peripheral lymph nodes to rapidly react to pathogens. These cells not only allow an immediate response, but also activate and amplify the adaptive immunity responses. This can be well illustrated by intestinal monocytes and macrophages in Crohn disease and ulcerative colitis^16,17^, neutrophils in systemic vasculitis^18^, and mast cells in psoriatic skin lesions^19^. IL17 has been implicated to be critical for the production of cytokines, neutrophil-related chemokine and antimicrobial peptides^20^. Wu *et al*. found that IL17 production is essential in neutrophil recruitment and activity in mouse lung defense against respiratory *Mycoplasma pneumoniae* infection^21^. In addition, serum levels of IgE, IL4, IL6 and IL10 were obviously higher in MPP patients in acute phase than those in the recovery phase^22^. Therefore, IL17, IL4 and IL10 may play roles in the immunological pathogenesis of MPP. Recently, interferon beta1 (IFNβ1) has been suggested as a possible immune regulatory cytokine of infection diseases^23^. In this study, we performed a retrospective study to investigate the expression of IL17A, IL4, IL10 and IFNβ1 in BALF by comparing their levels in mild MPP and severe MPP children.

The aim of the current study was to investigate the mRNA levels of different cytokines in BALF during the lower respiratory infection caused by MPP and identify biomarkers for children with severe MPP. BALF samples from 150 children were collected from our hospital, the levels of IL17A, IL4, IL10 and IFNβ1 were determined and related clinical information was analyzed. Children with bronchial foreign body were included as control group. BALF samples from 40 children from another hospital were collected and analyzed as a validation cohort.

## Results

### Basic information of the subjects from the First Hospital of Jilin University

A total of 114 children diagnosed with MPP and 36 children diagnosed with FB in the First Hospital of Jilin University from January 2015 to September 2016 were enrolled in the study (Table 1). All of the MPP children had positive results showing MP infection and had not fulfilled exclusive criteria. The fiberoptic bronchoscopy has been used to MPP children for the diagnosis and treatment of lobar pneumonia or segmental pneumonia. FB children had been included as control group; they received fiberoptic bronchoscopy for examination and removing bronchus foreign body. All of the BALF samples were collected before treatment. Total nucleated cell numbers in the BALF samples were within 5–10 × 10^6^ range. No significant difference had been found between mild MPP group and severe MPP group in the proportions of lymphocyte, macrophage, neutrophil and eosinophil [Additional file 1: Table S1]. This is a retrospective study, the MPP children were divided into mild MPP group and severe MPP group based on the clinical information collected and analyzed after September 2016, detailed criteria can be found in Methods.

**Table 1:** The basic information of the BALF samples from the First Hospital of Jilin University.

	Control	Mild MPP	Severe MPP
	(n = 36)	(n = 84)	(n = 30)
Gender (female/male)	13/23	36/48	14/16
Median age (range), years	1.67 (0.92–10.17)	4 (0.42–13)	6 (0.92–13)
Indication of bronchoscopy			
Diagnosis and remove of bronchus foreign body	22	0	0
Re-examination of bronchus foreign body	14	0	0
Diagnosis and treatment of lobar pneumonia	—	25	30
Diagnosis and treatment of segmental pneumonia	—	59	0
Timing of BALF collection			
Before treatment	36	84	30
After the treatment	0	0	0

Quantitative data with a non-normal distribution are presented as median (IQR).

### mRNA levels and predictive values of the cytokines in BALF of MPP children

**Figure 1:**
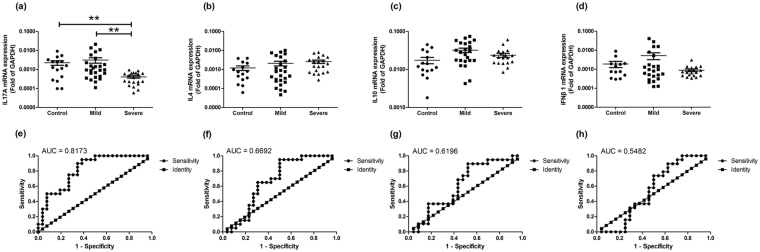
mRNA levels of different cytokines in BALF of children with MPP. (**a**) Relative expression levels of IL17A in control, mild MPP and severe MPP children. (**b**) Relative expression levels of IL4 in control, mild MPP and severe MPP children. (**c**) Relative expression levels of IL10 in control, mild MPP and severe MPP children. (**d**) Relative expression levels of IFNβ1 in control, mild MPP and severe MPP children. (**e**) Receiver operating characteristic (ROC) curve to evaluate the predicting value of IL17A in differentiating mild MPP from severe MPP. (**f**) ROC curve to evaluate the predicting value of IL4 in differentiating mild MPP from severe MPP. (**g**) ROC curve to evaluate the predicting value of IL10 in differentiating mild MPP from severe MPP. (**h**) ROC curve to evaluate the predicting value of IFNβ1 in differentiating mild MPP from severe MPP. The results of Benjamini-Hochberg p adjust are shown as: *p_adj_ < 0.05, **p_adj_ < 0.01, ***p_adj_ < 0.001.

mRNA levels of different cytokines had been observed in the BALF of MPP children and FB children. The relative expression levels of IL17A in severe MPP group were significantly lower than that of mild MPP group or that of control group (Fig. 1a). However, there was no significant difference in the relative expression levels of IL4 (Fig. 1b), IL10 (Fig. 1c), or IFNβ1 (Fig. 1d) between severe MPP group and mild MPP group.

To further explore the diagnostic ability of different cytokines for severe MPP, receiver operator characteristic (ROC) curves were made and the cutoff values with maximum sensitivities and specificities were determined. As shown in Fig. 1e and Table 2, the area under the receiver operating characteristic curve (AUC) of IL17A was 0.8173 (p = 0.00026). With a cutoff value of 0.00087, IL17A had a sensitivity of 0.9500, a specificity of 0.6154 in predicting severe MPP from mild MPP. With a positive predictive value of 0.6552 and a negative predictive value of 0.9412, IL17A may be used for differentiating children with severe MPP from those with mild MPP at the early stage of the disease (within 3 days after admission). However, AUC of IL4 was 0.6692 (Fig. 1f and Table 2, p = 0.05124), AUC of IL10 was 0.6196 (Fig. 1g and Table 2, p = 0.1806) and AUC of IFNβ1 was 0.5482 (Fig. 1h and Table 2, p = 0.5906). Based on these ROC analysis results, none of them can predict the severity of MPP.

**Table 2:** ROC curve analysis for predicting severe MPP in BALF of children.

Parameter	AUC	Cut off value	Sensitivity	Specificity	p value	95% confidence interval
IL17A	0.8173	0.00087	0.9500	0.6154	0.00026***	0.6966–0.9380
IL4	0.6692	0.00052	0.9500	0.5000	0.05124	0.5105–0.8280
IL10	0.6196	0.03166	0.9000	0.4783	0.1806	0.4478–0.7914
IFNβ1	0.5482	0.00140	0.8947	0.3750	0.5906	0.3719–0.7246

ROC: receiver operating characteristic; AUC: area under the ROC curve; Cut off value: the value on the ROC curve is closest to the upper right to take maximum sensitivity and specificity; p value: *p < 0.05, **p < 0.01, ***p < 0.001.

### Clinical characteristics and cytokine expression of the MPP children from the First Hospital of Jilin University

Children’s clinical information had been collected and analyzed after children’s discharge from the hospital (Table 3). According to the diagnostic criteria described in methods, children had been divided into mild group and severe group. 84 children were in the mild group (48 males, 36 females) with the median age of 4 (0.42–13) years, 30 children were in the severe group (16 males, 14 females) with the median age of 6 (0.92–13) years. The median age of the severe group was significantly older than that of the mild group, but no difference was found in gender distribution between the two groups. Comparing to mild MPP group, children in severe MPP group were more likely to become refractory MPP and receive glucocorticoid-treatment (p < 0.0001). In addition, severe MPP children were characterized by higher CRP (p < 0.05) and were more likely to have extrapulmonary manifestations (p < 0.05) comparing to mild MPP children. Significantly increased neutrophils (p < 0.05) and greatly decreased lymphocytes (p < 0.01) were found in the peripheral blood of severe group comparing to that of the mild group. However, no significant difference in blood eosinophil count was found between the two groups. These results need to be confirmed in a validation cohort.

**Table 3:** Clinical characteristics of the MPP children from the First Hospital of Jilin University.

	Mild MPP	Severe MPP	p value
	(n = 84)	(n = 30)	
Gender (female/male)	36/48	14/16	0.6701
Median age (range), years	4 (0.42–13)	6 (0.92–13)	<0.0001^***^
Fever duration, days	9.50 ± 7.80	10.96 ± 6.08	0.1229
Glucocorticoid (yes/no)	7/77	16/14	<0.0001^***^
Pleural effusion (yes/no)	1/83	9/21	<0.0001^***^
Extrapulmonary manifestations (yes/no)	1/83	3/27	0.0257^*^
Median CRP (range), mg/L	10 (0.01–191.20)	17.90 (2.50–196)	0.0478^*^
Peripheral blood cell count			
Blood leukocyte count (×10^9^ cells/L)	9.60 ± 4.67	10.21 ± 3.91	0.2209
Blood monocyte count (%)	8 (3–15)	7.50 (4–14)	0.3883
Blood neutrophil count (%)	55 (19–86)	68 (26–80)	0.0112^*^
Blood lymphocyte count (%)	33 (4–73)	19.50 (11–63)	0.0071^**^
Blood eosinophil count (%)	2 (0–5)	2 (0–5)	0.8536

Quantitative data with a normal distribution are presented as mean ± SD. Quantitative data with a non-normal distribution are presented as median (IQR).

*p < 0.05, **p < 0.01, ***p < 0.001.

**Figure 2:**
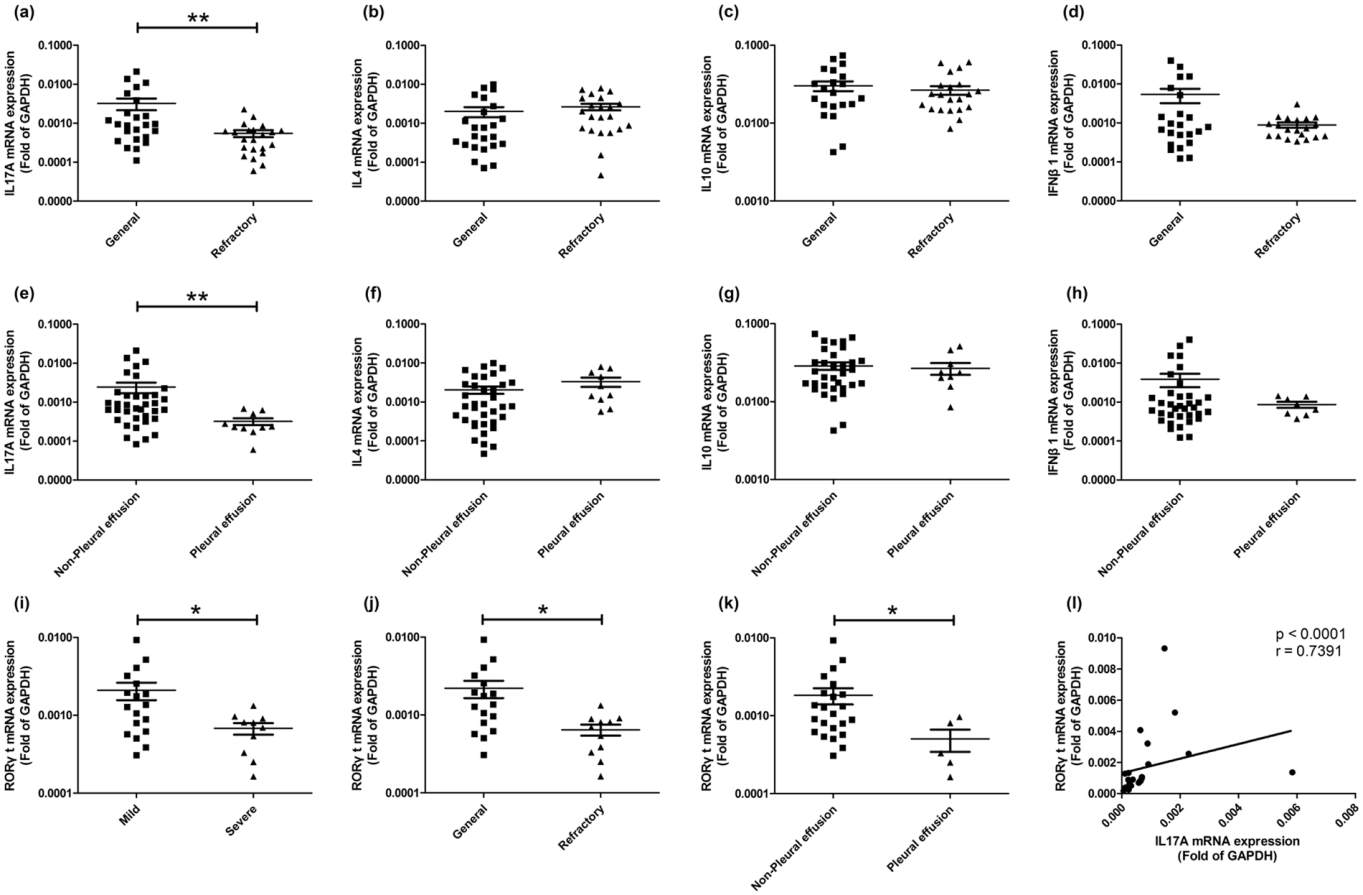
mRNA levels of different molecules in BALF of MPP children grouped with different clinical indicators. mRNA levels of IL17A (**a**), IL4 (**b**), IL10 (**c**) and IFNβ1 (**d**) in general MPP and refractory MPP children. mRNA levels of IL17A (**e**), IL4 (**f**), IL10 (**g**) and IFNβ1 (**h**) in MPP children with pleural effusion and MPP children without pleural effusion. (**i**) mRNA levels of RORγt in BALF of mild MPP and severe MPP children. (**j**) mRNA levels of RORγt in BALF of general MPP and refractory MPP children. (**k**) mRNA levels of RORγt in BALF of MPP children with pleural effusion and MPP children without pleural effusion. (**l**) The correlation of the mRNA levels of RORγt and IL17A in BALF of MPP children. The results of Benjamini-Hochberg p adjust are shown as: *p_adj_ < 0.05, **p_adj_ < 0.01, ***p_adj_ < 0.001.

The relationships between the mRNA levels of different cytokines in BALF and some clinical indicators were further observed. First, MPP children were divided into general group and refractory group based on the criteria described in Methods. As shown in Fig. 2a, the expression of IL17A in children with refractory MPP was significantly lower than that of children with general MPP. However, no significant difference was found in the expression of IL4, IL10 or IFNβ1 (Fig. 2b–d) between these two groups. Next, children with MPP were divided into two groups based on whether or not they had pleural effusion. As shown in Fig. 2e, the expression of IL17A in children with pleural effusion was obviously lower than that without pleural effusion. In contrast, no significant difference was found in the expression of IL4, IL10 and IFNβ1 (Fig. 2f–h) between MPP children with pleural effusion and that without pleural effusion.

Laboratory findings indicated elevated levels of CRP in MPP patients^10,11^. Table 3 showed increased levels of CRP in severe MPP group comparing to mild MPP group in this cohort. In order to explore the relationships between the mRNA levels of different cytokines and CRP, children were divided into three groups based on the different levels of CRP [Additional file 2: Figure S1]. No significant difference was found among different groups in the expression levels of IL17A, IL4, IL10 and IFNβ1.

In order to explore the possible reason that cause decreased IL17A in BALF of severe MPP children, the expression levels of RORγt were detected (Fig. 2i–l). As a transcriptional factor of IL17A, the levels of RORγt were significantly decreased in the BALF of severe MPP children comparing to that of mild MPP children. Moreover, greatly decreased RORγt had also been found in the BALF of refractory MPP children comparing to general MPP children and in BALF of MPP children with pleural effusion comparing to MPP children without pleural effusion. In addition, the relative expression levels of RORγt correlated significantly with the relative expression levels IL17A (p < 0.0001, r = 0.7391). Based on the published literatures, some cytokines including IL23, IL6 and TGFβ are involved in the process of IL17 expression^24–26^. Therefore, we detected the mRNA levels of IL23A, IL23R, IL6 and TGFβ by qPCR [Additional file 3: Figure S2]. Comparing to mild MPP group, significant difference had not been found in the severe MPP group in the mRNA levels of IL23A, IL23R, IL6 or TGFβ. Therefore, there is no evidence to support that the levels of IL23A, IL23R, IL6 or TGFβ in BALF of severe MPP children are lower than that of mild MPP children.

**Figure 3:**
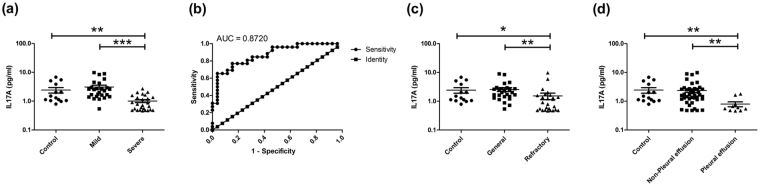
Protein levels of IL17A in BALF of MPP children. (**a**) Protein levels of IL17A in control, mild MPP and severe MPP groups. (**b**) ROC curve performed to evaluate the threshold of IL17A in differentiating mild MPP from severe MPP. Protein levels of IL17A reached area under the curve: 0.8720, p value: < 0.0001, cut off value: 1.366 pg/ml. It reached a sensitivity of 0.7692, a specificity of 0.8462, a positive predictive value of 0.8333 and a negative predictive value of 0.7857. (**c**) Protein levels of IL17A in control group, general MPP group and refractory MPP group. (**d**) Protein levels of IL17A in control group, MPP without pleural effusion group and MPP with pleural effusion group. The results of Benjamini-Hochberg p adjust are shown as: *p_adj_ < 0.05, **p_adj_ < 0.01, ***p_ad_j < 0.001.

Furthermore, in order to confirm that IL17A was decreased in BALF of severe MPP children, the protein levels of IL17A in the supernatant of BALF were detected by Enzyme linked immunosorbent assay (ELISA). As shown in Fig. 3a, the levels of IL17A in BALF were significantly lower in severe MPP group comparing to control group and mild MPP group. ROC curve was generated between the protein levels of IL17A in mild group and severe group. It reached area under the curve: 0.8720 (Fig. 3b) with a cutoff value of 1.366 pg/ml. The levels of IL17A had a sensitivity of 0.7692, a specificity of 0.8462 in predicting severe MPP from mild MPP. With a positive predictive value of 0.8333 and a negative predictive value of 0.7857, IL17A may be used for differentiating children with severe MPP from those with mild MPP at the earlier stage of disease. Interestingly, the levels of IL17A in refractory MPP group (Fig. 3c) were also greatly decreased comparing to that in general MPP group. Moreover, the protein levels of IL17A in BALF were significantly lower in MPP with pleural effusion group (Fig. 3d) comparing to that in MPP without pleural effusion group.

### Clinical characteristics and cytokine expression of MPP children from Changchun Children’s Hospital

To confirm that the levels of IL17A were decreased in BALF of severe MPP children, a total of 40 children diagnosed with MPP in Changchun Children’s Hospital from November 2016 to December 2016 were enrolled in the study as a validation cohort (Table 4). Clinical information had been collected after December 2016; MPP children had been divided into mild MPP group and severe MPP group based on the criteria described in the Methods. Mild MPP group included 20 children (12 males, 8 females) with the median age of 6.5 (2.20–9.92) years; severe MPP group included 20 children (14 males, 6 females) with the median age of 5 (1.67–8.75) years. Comparing to mild MPP group, children in severe MPP group were more likely to become refractory MPP and receive glucocorticoid treatment (p < 0.0001). These results were similar to the findings in the MPP children from the First Hospital of Jilin University (Table 3). However, no significant difference was found in the median age, median CRP and peripheral blood cell count between mild MPP group and severe MPP group. Therefore, the significant difference found in median age, median CRP and peripheral blood cell count between the two groups in the children from the First Hospital of Jilin University had not been confirmed.

**Table 4:** Clinical characteristics of the MPP children from Changchun Children’s Hospital.

	Mild MPP	Severe MPP	p value
	(n = 20)	(n = 20)	
Gender (female/male)	8/12	6/14	0.5233
Median age (range), years	6.50 (2.20–9.92)	5 (1.67–8.75)	0.3844
Fever duration, days	4.55 ± 1.57	8.45 ± 2.72	<0.0001***
Glucocorticoid (yes/no)	2/18	17/3	<0.0001***
Pleural effusion (yes/no)	0/20	4/16	—
Extrapulmonary manifestations (yes/no)	0/20	0/20	—
Median CRP (range), mg/L	21.50 (2–180)	19.50 (7–140)	0.8392
Peripheral blood cell count			
Blood leukocyte count (×10^9^ cells/L)	8.85 ± 2.45	10.79 ± 5.05	0.1555
Blood monocyte count (%)	5.40 (0.37–10.10)	7.03 (1.30–11.14)	0.0857
Blood neutrophil count (%)	58.60 (10–80.22)	62.57 (20.30–84.50)	0.1941
Blood lymphocyte count (%)	26.30 (11.50–52.60)	24 (13–73.6)	0.9031
Blood eosinophil count (%)	2 (0–5)	2 (0–5)	0.6801

Quantitative data with a normal distribution are presented as mean ± SD. Quantitative data with a non-normal distribution are presented as median (IQR).

*p < 0.05, **p < 0.01, ***p < 0.001.

**Figure 4:**
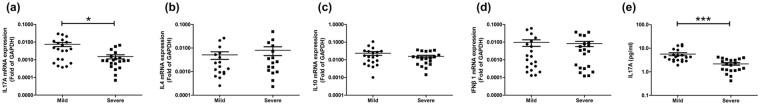
mRNA levels of different cytokines and protein levels of IL17A in BALF of MPP children from Changchun Children’s Hospital. (**a**) mRNA levels of IL17A in BALF of mild MPP and severe MPP children. (**b**) mRNA levels of IL4 in BALF of mild MPP and severe MPP children. (**c**) mRNA levels of IL10 in BALF of mild MPP and severe MPP children. (**d**) mRNA levels of IFNβ1 in BALF of mild MPP and severe MPP children. (**e**) Protein levels of IL17A in mild MPP and severe MPP children. The results of Benjamini-Hochberg p adjust are shown as: *p_adj_ < 0.05, **p_adj_ < 0.01, ***p_adj_ < 0.001.

Interestingly, similar results regarding the mRNA levels of different cytokines had been found in the BALF samples from this validation cohort. As shown in Fig. 4a, the relative expression levels of IL17A in severe MPP group were significantly lower than that of mild MPP group. However, no significant difference had been found in the relative expression levels of IL4 (Fig. 4b), IL10 (Fig. 4c) or IFNβ1 (Fig. 4d) between the two groups. Furthermore, the protein levels of IL17A in the supernatant of BALF were detected by ELISA. As shown in Fig. 4e, the levels of IL17A in BALF were significantly lower in severe MPP group comparing to mild MPP group. Therefore, the significantly decreased IL17A levels found in the BALF of severe MPP children comparing to that of mild MPP children in the First Hospital of Jilin University had been confirmed in this validation cohort.

## Discussion

MPP is usually found in children and young adults to be a self-limited process, but sometimes severe MPP happens with serious complications. Early distinction of severe MPP from mild MPP is very important as to avoid delay of effective treatment. Our current study observed mRNA levels of different cytokines in the BALF of MPP children and analyzed data with different clinical manifestations. The expression levels of IL17A in BALF are significantly decreased in severe MPP comparing to mild MPP and control, but the expression levels of IL4, IL10 and IFNβ1 are not significantly changed in severe MPP comparing to that of mild MPP. Furthermore, greatly decreased levels of IL17A were also found in refractory MPP group and MPP with pleural effusion group. Our data suggest that expression levels of IL17A in BALF have diagnosis value of the severity of MPP, but expression levels of IL4, IL10 and IFNβ1 do not have this diagnosis value. We also studied the levels of IL17A in the BALF samples of a separate cohort from the second medical center and validated the original observations.

The clinical characteristics were collected and analyzed after children were discharged from hospital. Results from one medical center showed that severe MPP children had significantly increased CRP and decreased lymphocyte numbers comparing to mild MPP children. However, similar changes had not been found in the children from another medical center. Early identification of severe MPP based on the clinical information is still difficult. The current study showed that IL17A expression in BALF was significantly decreased in severe MPP children comparing with mild MPP children; ROC analysis revealed that the presence of high levels of IL17A in BALF indicated a low probability of severe MPP. Therefore, decreased IL17A in BALF might be a useful biomarker of severe MPP, which is helpful to early diagnosis and screening children of severe MPP. We found some links between IL17A expression and the clinical characteristics of severe MPP. Higher IL17A expression in BALF is correlated with general MPP and MPP without pleural effusion; lower IL17A expression in BALF is correlated with refractory MPP and MPP with pleural effusion. Similar to our results, it has been reported that MPP children with pleural effusion may have a more severe form of MPP with longer hospital stay and more commonly usage of corticosteroid comparing to those without pleural effusion^5^. We put forward the hypothesis that low IL17A levels in BALF may suggest severe MPP in children and more likely to become MPP with pleural effusion and refractory MPP.

The immunological pathogenesis of severe MPP is still unclear. Decreased IL17A in BALF of severe MPP children comparing to mild MPP children may be involved in the pathogenesis of severe MPP. Many publications have reported a functional link between IL17A and infection^27^. The major populations of lymphocytes that secrete IL17 include TCRγδ+ γδ T cells, CD4+TCRαβ+TH17 cells, CD3- ILC3s, TCR+NK1.1+ NKT cells and TCR-NK1.1+ NK cells^20^. Bacteria, fungi, viruses and parasites share the capacity to induce a major production of IL17 during the early innate response^28–30^. IL17 play an essential role in the recruitment of neutrophils to the local environment of infection^31^. In epithelia, such as in the small intestine, IL17 is important for the prevention of microbial invasion^32^: First, it acts with IL22 to induce the expression of AMPs. Second, IL17 has an important role in the recruitment of phagocytic cells to prevent the spread of infection. Third, IL17 is important for the generation of IgA, which offers lifelong protection against invading microorganisms. At last, successful immunity with minimal immunopathology is often achieved by a balance of IL17 and IFNγ. In the respiratory tract, IL17A is involved in protection against K. pneumoniae, which causes pulmonary infections in mice. In the absence of IL17RA, which prevents responses from both IL17A and IL17F, immunity to K. pneumoniae is curtailed, with reduced expression of chemokines and G-CSF, thereby leading to reduced neutrophil recruitment, increased bacterial burden and increased mortality^33^. Recent progress shows importance of IL17A in both innate and acquired immunity against infections, it is considered as an important inflammatory mediator that is critical in the protection from pneumococcal colonization in airways^34,35^. In agreement with these observations, we found decreased IL17A levels in children with severe MPP, which may indicate that the immune-regulatory function of IL17A is insufficient in BALF of these children. Insufficient IL17A may be involved in the pathogenesis of severe MPP.

On the other hand, no significant difference was found in the levels of IL4, IL10 and IFNβ1 in severe MPP group comparing to that in mild MPP group. However, in several publications, IL4 and IL10 are found to be significantly increased in mice that are repeatedly infected with M. pneumonia^36,37^. IL4 levels in BALF are significantly higher in patients with MPP than in control participants^38^. IL10 in refractory MPP group is significantly higher than those in general MPP group^39^. Conversely, decreased IL10 is found in the peripheral blood of children with severe MPP by another research group^40^. These results suggest that IL4 and IL10 may play roles in the pathogenesis of MPP. IFNβ has been shown to be an effective therapy for multiple sclerosis (MS). Recent findings demonstrate a regulatory role for IFNβ in autoimmune inflammation in mice model of MS and experimental autoimmune encephalomyelitis (EAE)^23^. Another study shows IFNβ-treatment of T cells result in reduced production of IL17 and increased production of IL10^41^.

Significantly increased neutrophils have been found in severe MPP comparing to mild MPP in the peripheral blood in the cohort from the First Hospital of Jilin University. However, this results have not been confirmed in the cohort from Changchun Children’s Hospital. In order to further explore the local changes, we have counted nucleated cells in the BALF of the MPP patients. No significant difference between mild MPP group and severe MPP group has been found in the proportions of lymphocyte, macrophage, neutrophil and eosinophil. Therefore, there is not enough evidence to support that neutrophils are increased in severe MPP children. However, Guo *et al*. reported that comparing to mild group, the percentage of neutrophils were increased in children with severe MPP (p < 0.01)^42^. Another group reported that the percentage of neutrophil in BALF was higher in the group of patients with high levels of *M*. *pneumoniae* DNA than in those with low levels of *M*. *pneumoniae* DNA (p < 0.05)^43^. As we know, various cells are involved in the inflammation response, such as macrophages, neutrophils, lymphocytes as well as bronchial epithelial cells. Published literatures reported that macrophages rather than neutrophils are essential for the clearance of *M*. *pneumoniae* from the lungs^44^. On the contrary, neutrophil accumulation might lead to “hyperinflammation” due to matrix metalloproteinase-9 (MMP-9), myeloperoxidase (MPO) and neutrophil elastase (NE) release^43^. Further study will be needed to prove the changes and pathological role of neutrophils in severe MPP.

Some limitations of the current study are worth discussing. First, relationship between IL17A and other cytokines in BALF of MPP children has not been examined. IL4 deficient mice show a marked increase in IL17A concentration with inhibited eosinophil recruitment^45^. Significant difference of the blood eosinophil cell-count was not found between severe MPP group and mild MPP group in this study. However, our results show increased IL4 levels and decreased IL17A levels in severe MPP comparing to mild MPP. It is still unclear if the increased levels of endogenous IL17A is related to the decreased levels of IL4 expression in MPP children. The presence of IL17 and IFNγ at inflammatory sites and the specific roles of these cytokines in immunopathology have been widely reported^46^. A balance of IL17 and IFNγ is important to achieve successful immunity with minimal immunopathology^47^. Acting in synergy with TNF, IL17 is a powerful inflammatory signal that results in the rapid recruitment and sustained presence of neutrophils^32^. It may be interesting to further explore the relationship between IL17 and IL4, IL17 and IFNγ, IL17 and TNF in severe MPP infection. Second, further studies will be needed to explore the mechanism of the lower IL17A expression in BALF of severe MPP children. RORγt is an important transcription factor of IL17^14,48^. It is expressed on Th17 cell, lymphoid tissue inducer (LTi) and IL17 producing ILC3 (ILC17)^17,49^. Other RORγt+ cells also include NKT cell and γδ T cell^20^. Our results show significantly decreased expression levels of RORγt in severe MPP group comparing to mild MPP group. In addition, significant positive correlations are also found between the expression levels of RORγt and IL17A in MPP children. Further study will be needed to support the hypothesis that decreased IL17A levels in BALF may be caused by a decrease in RORγt levels.

In summary, the current study on BALF samples from two medical centers find significantly decreased IL17A in severe MPP children comparing to mild MPP children. These results may suggest that decreased IL17A in BALF is a valuable biomarker for severe MPP children, which help to distinguish severe MPP from mild MPP in the early stage of disease. However, measurement of IL4, IL10 and IFNβ1 in BALF has poor diagnostic value in the severity of MPP. Further studies will be needed to explore the pathogenesis role of IL17A in children with severe MPP, which could provide significant insights into severe MPP therapy in the future.

## Methods

### Individuals and samples

BALF samples of 150 children admitted to the First Hospital of Jilin University and 40 children admitted to Changchun Children’s Hospital were obtained and their clinical information were retrospectively collected. The diagnosis of MP infection was based on serologic testing and confirmed by polymerase chain reaction (PCR) tests of nasopharyngeal secretions. The indications of bronchoscopy is based on the 2009 edition of the Guide to pediatric bronchoscopy^50^, children with mild MPP were only chosen when they had segmental pneumonia or lobar pneumonia. BALF samples were only collected from children with acute MPP before treatment.

The mild and the severe community-acquired pneumonia were defined based on the criteria described^51–53^. Mild was defined as respiratory rate <70 breaths/min at age <3 years old or respiratory rate <50 breaths/min at age ≥3 years old, normal food-intake, no dehydration. Severe was defined as respiratory rate ≥70 breaths/min at age <3 years old or respiratory rate >50 breaths/min at age ≥3 years old (excluding the reasons of fever and cry), cyanosis, flaring of the nares, marked retractions, anorexia and dehydration; or MPP combined with pleural effusion, lung necrosis/lung abscess and other complications in lung; or MPP combined with dysfunction of other systems. Refractory MPP group was defined as continuous fever >38.5 °C or no improvement in lung imaging after the effective application of macrolide drugs for more than 1 weeks^6,7^. By measuring the maximum thickness of fluid between the visceral and parietal pleurae on a CT scan, pleural effusion was defined as fluid thickness >5 mm^54^. The control group consisted of 36 children with airway foreign body (FB) aspiration that had no respiratory tract illness in the previous 6 weeks.

All children with other respiratory tract infections and tuberculosis were excluded based on following tests: protein purified derivative (PPD), blood cultures, pleural effusion cultures, nasopharyngeal aspirate/swab cultures, nasopharyngeal aspirate/swab for virus antigens detection and serology for *Chlamydia pneumoniae* and *Legionella pneumophila*. Children who received corticosteroids before admission or had underlying diseases such as asthma, recurrent respiratory tract infection, chronic cardiac disease, rheumatic diseases and immunodeficiency were also excluded.

### Brochoscopy and bronchoalveolar lavage

Following the guidelines described previously^50,51,55^, flexible fiber optic bronchoscopy with bronchoalveolar lavage was performed within 3 days after the admission. BALF was gently aspirated, collected and centrifuged at 1500 rpm for 5 minutes at 4 °C within 1 hour after collection. The pellet was resuspended in TRIzol reagent (Invitrogen, Carlsbad, CA, USA) and stored in −80 °C freezer. The supernatant was divided into 250 µl aliquots and stored in −20 °C freezer.

### Enzyme-linked immune sorbent assay (ELISA)

The concentrations of IL17A were measured using Human IL17A ELISA Kit (eBioscience) and Human IL17A High Sensitivity ELISA Kit (eBioscience) according to the manufacturer’s instructions.

For the Human IL17A ELISA Kit, the plate was first sealed with 100 µl/well of Capture antibody in 1X Coating buffer and incubated overnight at 4 °C. Second, the plate was washed 3 times with Wash buffer and blocked with 200 µl/well of 1X ELISA diluents at room temperature for 1 hour. After aspiration and wash, BALF supernatant samples, standard dilutions of human IL17A and blank were added to the plate as 100 µl/well. After adding the samples, plate was incubated at room temperature for 2 hrs on a microplate shaker setting at 400 rpm. Next, the plate was washed and incubated with Detection antibody, Streptavidin-HRP and TMB substrate solution in sequential order following the instructions. 100 µl/well Stop solution was added when the highest standard has developed a dark blue color. For the Human IL17A High Sensitivity ELISA Kit, wash the microwell strips twice with Wash buffer. Add Standard dilutions of human IL17A to the plate 100 µl/well. Add 100 µl of Sample diluent to the blank wells. Add 50 µl of Sample diluent and 50 µl sample to each sample well. Add 50 µl of Biotin-conjugate to all wells. Cover with an adhesive film and incubate at room temperature overnight in the dark. Remove adhesive film, empty wells and wash microwell strips 6 times. Add 100 µl of diluted Streptavidin-HRP to all wells. Cover with an adhesive film and incubate at room temperature for exactly 1 hour on a microplate shaker in the dark. Remove adhesive film, empty wells and wash microwell strips 6 times. Add 100 µl of Amplification solution I to all wells. Cover with an adhesive film and incubate at room temperature for exactly 15 minutes on a microplate shaker in the dark. After aspiration and wash, add 100 µl of Amplification solution II to all wells. Cover with an adhesive film and incubate at room temperature for exactly 30 minutes on a microplate shaker in the dark. Remove and wash microwell strips 6 times. Pipette 100 µl of TMB Substrate solution to all wells. Incubate the microwell strips at room temperature for about 10–20 minutes in the dark. 100 µl/well Stop solution was added when the highest standard has developed a dark blue color.

For both Human IL17A ELISA Kit and Human IL17A High Sensitivity ELISA Kit, absorbance of the plate was read on the Synergy H1 Hybrid Reader (Biotek, Winooski, VT, USA) using 450 nm as the primary wave length. Standard curve was generated from the readings of the diluted standards. Sample concentrations were calculated based on their absorbance comparing to the standard curve. All of the samples were tested twice and the results were averaged concentrations.

### Real-time quantitative PCR (qPCR)

qPCR was performed as described previously^56^. Briefly, total RNA was extracted from BALF cells using TRIzol reagent (Invitrogen, Carlsbad, CA, USA). Extracted RNA was reverse-transcribed using the Prime Script RT Reagent Kit (TAKARA, Kyoto, Japan). cDNA was amplified using the Fast Start Universal SYBR Green Master (Roche Diagnostics GmbH, Mannheim, Germany) and analyzed with quantitative PCR using specific primers (synthesized by Sangon Biotech, Shanghai, China) on the Applied Biosystems Step one plus instrument (Step one software 2.2). Each sample was tested twice; every Ct value was the average of the results from two wells. Glyceraldehyde-3-phosphate dehydrogenase (GAPDH) was selected as the reference gene. The method of 2^−ΔΔCT^ was used to analyze the real-time PCR data expressed as the fold-change relative to the average value of the GAPDH^57,58^.

### Ethics approval and consent to participate

Ethical approval for the study was received from the Institutional Medical Ethics Review Board of the First Hospital of Jilin University (reference number: 2015-238) in compliance with the Declaration of Helsinki. Ethical approval for the validation cohort was received from the Institutional Medical Ethics Review Board of Changchun Children’s Hospital (reference number: 2016-010) in compliance with the Declaration of Helsinki. Care givers of all children provided written informed consents.

### Statistical Analysis

Statistical analyses were performed using Graphpad 5.0 or SPSS software (version 22.0). Chi-squared tests were used to compare categorical data. The comparisons were carried out with the Mann-Whitney U test. Receiver operating characteristic (ROC) analysis was conducted to examine the diagnostic ability of candidate cytokines for predicting the severity of MPP. The Positive predictive value and Negative predictive value were calculated with MedCalc 15.1. The nonparametric Spearman rank correlation test was applied for the correlation studies. Statistical significance was defined as p < 0.05. For the reanalyzed data sets, the related p values have been adjusted by Benjamini-Hochberg procedure (BH)^59^ using a statistical analysis software (R programming language 3.1.1). After adjustment, adjusted p value has been expressed as p_adj_, statistical significance had been defined as p_adj_ < 0.05.

### Availability of data and material

The datasets used and/or analyzed during the current study are available from the corresponding author on reasonable request.

## Electronic supplementary material

Dataset 1
