# Supplementary material for: Interleukin 17A as a good predictor of the severity of Mycoplasma pneumoniae pneumonia in children
Source: Sci Rep. 2017 Oct 11;7:12934. doi: 10.1038/s41598-017-13292-5 (PMC5636901; doi:10.1038/s41598-017-13292-5)

**Supplementary Information**

**Interleukin 17A as a good predictor of the severity of *Mycoplasma pneumoniae* pneumonia in children**

Mingyue Yang^1#^, Fanzheng Meng^2#^, Kuo Wang^1^, Man Gao^2^, Ruihua Lu^2^, Mengyao Li^3^, Fangxing Zhao^4^, Lijuan Huang^4^, Yining Zhang^2^, Genhong Cheng^1,5^, Xiaosong Wang^1*^

1. Department of Translational Medicine, the First Hospital of Jilin University, Changchun, Jilin, 130021, China.

2. Department of Pediatrics, the First Hospital of Jilin University, Changchun, Jilin, 130021, China.

3. The Bethune Medical School of Jilin University, Changchun 130021, China.

4. Department of Respiratory, Changchun Children's Hospital, Changchun, 130051, China.

5. Department of Microbiology, Immunology and Molecular Genetics, University of California Los Angeles, Los Angeles, CA 90095, USA.

**^＃^ These authors contributed equally to this work.**

* **Correspondence author:**

Dr. Xiaosong Wang

Department of Translational Medicine, the First Hospital of Jilin University

Changchun, Jilin, 130021, China

Tel: +86 138 4498 9650

E-Mail: [xiaosongwang@jlu.edu.cn](mailto:xiaosongwang@jlu.edu.cn)

**Additional file 1: Table S1. Nucleated cell count in the BALF of the MPP children**

|  | **n** | **Macrophage** | **Lymphocyte** | **Neutrophil** | **Eosinophil** |
| --- | --- | --- | --- | --- | --- |
| **Mild MPP** | 20 | 77.04 ± 8.79 | 10.38 ± 8.79 | 10.10 ± 7.88 | 0.20 ± 0.41 |
| **Severe MPP** | 13 | 72.41 ± 11.82 | 14.43 ± 9.11 | 12.06 ± 11.19 | 0.27 ± 0.47 |
| ***t*** | - | 1.2120 | 1.2670 | 0.5514 | 0.4327 |
| ***p*** | *-* | 0.2396 | 0.2169 | 0.5878 | 0.6704 |
| **Quantitative data with a normal distribution are presented as mean ± SD.** | | | | | |

**Additional file 2: Figure S1. mRNA levels of cytokines in MPP children with different levels of CRP.** The expression levels of IL17A (a), IL4 (b), IL10 (c) and IFNβ1 (d) in patients with different levels of CRP. All of the p values > 0.05.


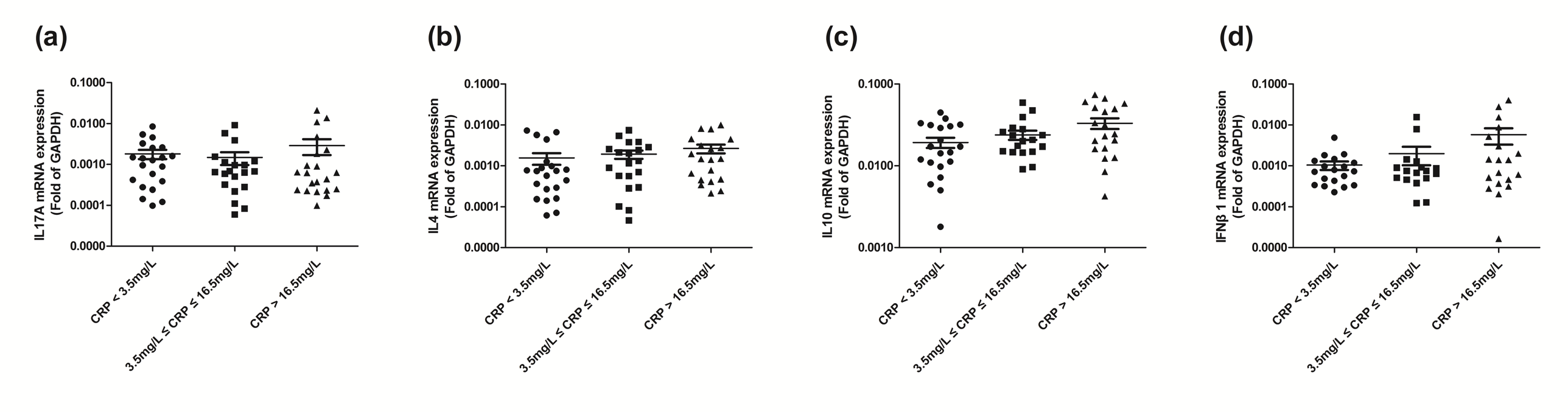


**Additional file 3: Figure S2. mRNA levels of different cytokines in BALF of children with MPP.** (a) Relative expression levels of IL23A in control, mild MPP and severe MPP children. (b) Relative expression levels of IL23R in control, mild MPP and severe MPP children. (c) Relative expression levels of IL6 in control, mild MPP and severe MPP children. (d) Relative expression levels of TGFβ1 in control, mild MPP and severe MPP children. All of the p values > 0.05.


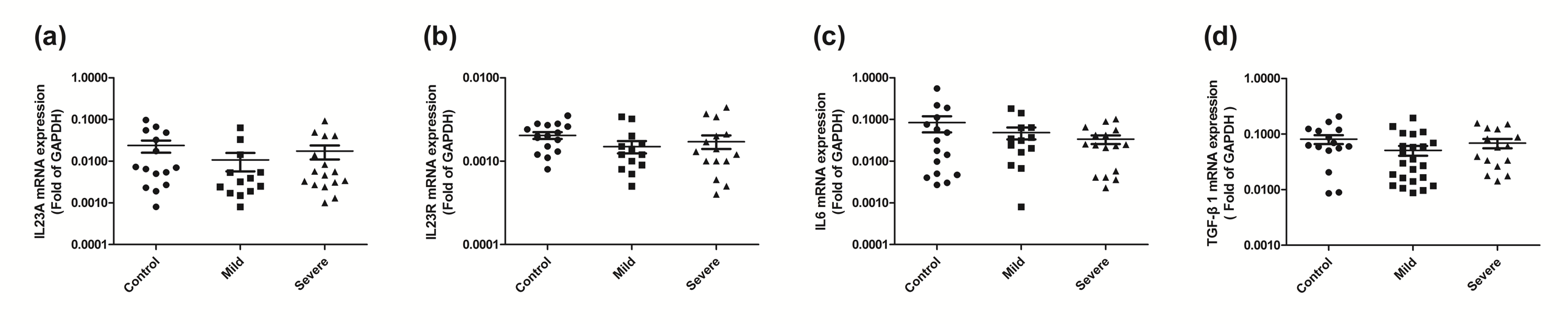

Supplement: Supplementary file 1 — Dataset 1 [file 41598_2017_13292_MOESM1_ESM.doc]
